# Gender equity in radiology and radiology research: a survey by the European Society of Radiology

**DOI:** 10.1186/s13244-026-02281-w

**Published:** 2026-05-06

**Authors:** Anna D’Angelo, Elisabetta Giannotti, Minerva Becker, Chiara Giraudo, Camilla Panico, Anagha P. Parkar, Anouk van der Hoorn, Pascal A. T. Baltzer, Diana Giannarelli, Ritse M. Mann, Marion Smits, Paola Clauser, European Society of Radiology (ESR)

**Affiliations:** 1 Department of Diagnostic Imaging, Oncological Radiotherapy and Hematology, Fondazione Policlinico Universitario “A. Gemelli” IRCCS, Rome, Italy; 2 Cambridge Breast Unit, Cambridge University Hospitals, NHS Foundation Trust, Cambridge, UK; 3 Unit of Head and Neck and Maxillo-facial Radiology, Division of Radiology, Diagnostic Department, University Hospitals of Geneva, Geneva, Switzerland; 4 Department of Cardiac, Thoracic, Vascular Sciences and Public Health–DCTV, University of Padova, Padova, Italy; 5 Radiology Department, Haraldsplass Deaconess Hospital, Bergen, Norway; 6 Department of Radiology, Medical Imaging Center (MIC), University Medical Center Groningen, Groningen, The Netherlands; 7 High-field MR Center, Department of Biomedical Imaging and Image-Guided Treatment, Medical University of Vienna, Vienna, Austria; 8 Biostatistics Unit, Fondazione Policlinico Universitario “A. Gemelli” IRCCS, Rome, Italy; 9 Department of Medical Imaging, Radboud University Medical Centre, Nijmegen, The Netherlands; 10 Department of Radiology, The Netherlands Cancer Institute, Amsterdam, The Netherlands; 11 Department of Radiology & Nuclear Medicine, Erasmus MC–University Medical Center Rotterdam, Rotterdam, The Netherlands; 12 European Society of Radiology, Am Gestade 1, Vienna, Austria

**Keywords:** Radiology, Gender equity, Job satisfaction, Work-life balance, Leadership

## Abstract

**Objectives:**

Gender equity in medicine remains a topic of increasing attention. The aim was to investigate if gender influences the radiology profession, with a focus on career progression, leadership roles, work-life balance, research activity and perceived barriers.

**Materials and methods:**

An anonymous online survey consisting of 22 questions was distributed by the European Society of Radiology (ESR) to its members between October and December 2024. The survey covered demographics, work schedules, family responsibilities, career development, leadership roles, research involvement, and perceived personal experiences. Quantitative data were analyzed using descriptive statistics, chi-square test, and rate differences with confidence intervals. Open-ended responses were explored qualitatively using thematic analysis.

**Results:**

Among 830 respondents, 657 completed the questionnaire (63.3% female, 35.3% male, 1.3% others). Women more frequently reported caregiving responsibilities beyond childcare (4.1% vs 3%), longer parental leave (46.2% vs 21.5%), and experiences of harassing behaviors at work. Men held a higher proportion of leadership roles (33.2% vs 25.2%). Respondents involved in research were more likely to work > 30% extra hours (47.2% vs 29.0%). Although research activity rates were similar across genders, women more often reported barriers to attending conferences and a lack of protected research time. Career fulfillment increased with age among men but decreased among women. Gender was considered a career disadvantage by 44.5% of women versus 9.5% of men.

**Conclusion:**

The survey reveals perceived gender disparities in radiology, particularly in leadership access, work conditions, and career satisfaction. Addressing structural barriers and promoting supportive workplace policies are essential to achieving true gender equity in the field.

**Critical relevance statement:**

Despite improvements in the last few decades, gender inequity remains present in radiology. Variability between geographical regions suggests that key critical areas can be addressed to promote improvement and support a more equitable professional environment.

**Key Points:**

- Perceived gender disparities in radiology are present across career progression, leadership roles, and work-life balance.
- Women were significantly more likely than men to perceive gender as a career disadvantage (44.5% vs 9.5%; *p* < 0.0001 35.0% [95% CI: 28.9 to 41.1]). They reported slightly higher caregiving responsibilities and longer parental leave.
- Structural inequalities impact gender equity in radiology, requiring targeted institutional and cultural changes.

**Graphical Abstract:**

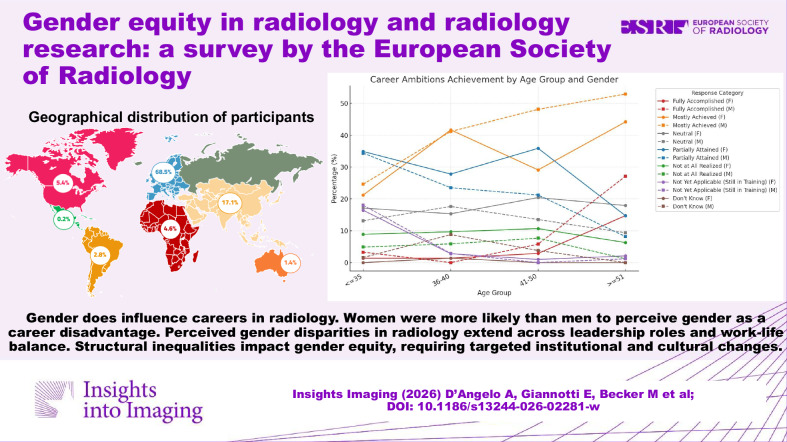

## Introduction

Over the past decade, discussions on equity, diversity, and inclusion (EDI) have gained momentum in radiology and other medical specialties, driven by societal movements, institutional initiatives, and growing awareness of disparities in access, representation, and opportunity [1–3]. Key EDI factors include gender distribution, leadership, and authorship, as well as the impact of ethnicity and origin on careers. Marked cross-country disparities highlight the need for targeted EDI analyses in European radiology [4]. Despite increasing attention, data on EDI within European radiology remain limited and fragmented, with most of the current evidence coming from non-European countries [5]. Some European medical societies have been implementing initiatives to increase evidence and awareness on these topics [6].

The European Society of Radiology (ESR), with its large and diverse membership base, represents a unique opportunity for multifaceted data collection [7]. The survey explores the perceived influence of gender on career progression, workplace interactions, and professional experiences in radiology research, while identifying key areas for improvement to support greater gender balance and inclusivity.

## Methods

### Survey design and distribution

The study received approval from the Institutional Review Board of the Fondazione Policlinico Universitario “A. Gemelli,” Rome, Italy (Lazio Area 3, ID 6866, 04.10.2024).

The survey was developed by six radiologists (2 males and 4 females; aged 37–45 years) working in academic hospitals across Europe, assisted by an experienced biostatistician. When possible, questions were based on existing surveys and articles [8–10]. The survey examined gender equity in radiology across demographics, family responsibilities, work conditions, career advancement, perceived bias, and overall satisfaction. The final version was reviewed and approved by the ESR Research Committee and by the Board of Directors.

The final 22-question survey, including multiple-choice and open-ended responses, was tested for clarity by 10 radiologists and 5 non-radiologists (medical doctors or radiographers), and refined accordingly. It was implemented in English using SurveyMonkey (www.surveymonkey.com) and distributed via email by the ESR to all its members between 1 October 2024 and 9 December 2024. Data were collected anonymously. The initial dissemination email was sent to 106,775 recipients, followed by a reminder to 60,856 non-openers on 4 December. The questionnaire is included in the Supplementary Material (SM).

### Data analysis

Data were summarized as absolute counts and percentages. Age was reported as median and range and compared between groups using the Mann–Whitney U test.

In order to ensure subgroups with a sufficient size for sub-analyses, countries were grouped following the United Nations geographical regions [11].

Cross-tabulations were performed with gender, age and geographical regions to analyze associations between items and these three factors; the chi-square test was used for determining statistical significance of differences. Group comparisons were assessed using absolute rate differences with 95% confidence intervals (CIs) to evaluate statistical significance and the estimate precision. Due to the low number of non-binary participants, subgroup analysis included only female or male participants. A two-sided *p*-value < 0.05 was considered statistically significant. Statistical analyses were performed using IBM-SPSS Statistics for Windows, v.28.0 (IBM Corp).

Open-ended responses were analyzed using ATLAS.ti (www.atlasti.com, Scientific Software Development GmbH).

## Results

### General description

A total of 830 responders participated in the survey. Of those, 657 completed the entire questionnaire (79.2%). Partial responses were excluded.

**Fig. 1:**
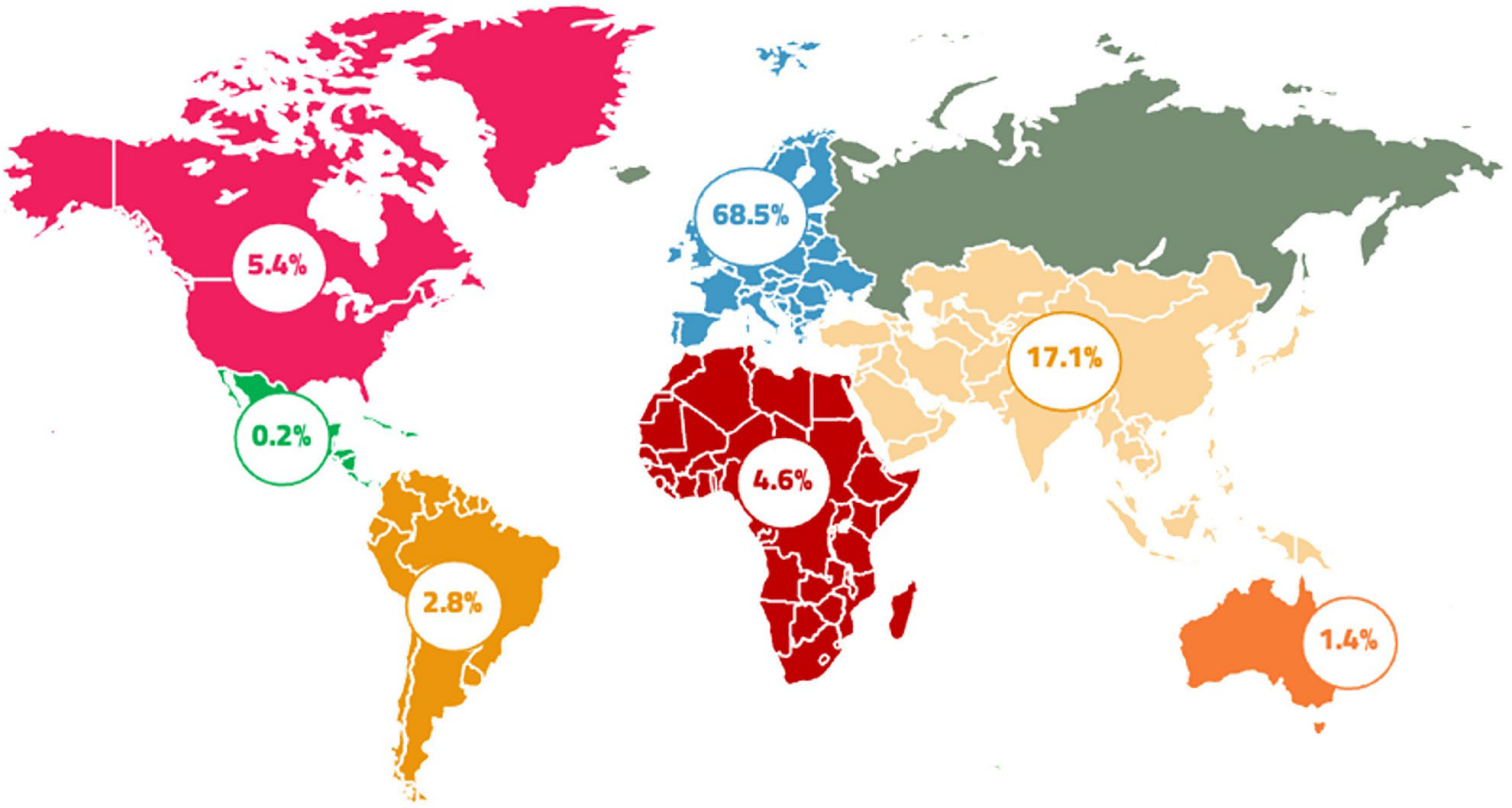
Geographical distribution of participants. Most participants lived and worked in Europe (68.5%) or Asia (17.1%). Other represented regions were North America (5.4%), Africa (4.6%), South America (2.8%), Oceania (1.4%), and Central America (0.2%)

Among the respondents, 63.3% identified as female (*n* = 416), 35.3% as male (*n* = 232), and 1.3% as other (*n* = 9) (4 “non-binary,” 2 “prefer not to disclose,” and 3 “other”). The median age of participants was 41 years (range: 21–77), higher in male respondents (43 years, range: 22–77) than in females (40 years, range: 21–75; *p* < 0.001). The geographical distribution of participants is reported in Fig. 1. A complete list of participating countries is provided in Table S1 in the SM.

Most respondents were employed in University Hospitals or research centers (64.7%), followed by Public Hospitals (29.2%), Private Hospitals (21.0%), or other types of healthcare facilities (5.6%).

Most of the participants were clinical radiologists (31.3%), followed by radiologists in leadership positions (26.6%), and radiologists in training/fellows (17.7%) (Table S2 in SM). Subspecialities of radiology practice are shown in Table 1.

**Table 1:** Subspecialty/area of practice

Subspecialty/area of practice	*N*	%
Breast radiology	125	19.0%
Cardiac and vascular radiology	69	10.5%
Chest radiology/thoracic imaging	48	7.3%
Emergency radiology	33	5.0%
Gastrointestinal and abdominal radiology	109	16.6%
Head and neck radiology	33	5.0%
Interventional radiology	71	10.8%
Medical imaging informatics	6	0.9%
Musculoskeletal radiology	55	8.4%
Neuroradiology	103	15.7%
Oncologic imaging	61	9.3%
Pediatric radiology	54	8.2%
Urogenital radiology	59	9.0%
General radiology/no specific subspecialty	149	22.7%

General radiology was the most common area of practice (23.0%), followed by breast (19.0%), gastrointestinal/abdominal (16.5%), and neuroradiology (15.9%)

Overall, 65.4% of respondents were engaged in research activities, with no significant gender difference (68.1% men vs 63.9% women, *p* = 0.28).

Among the 648 respondents, 69.1% studied and currently worked in their birth country (68.8% of women, 69.8% of men, *p* = 0.84, 1.1% [95% CI: 0.0–8.5]), 11.0% studied in their birth country and then moved abroad (12.7% of women, 7.8% of men, *p* = 0.069, 5.0% [95% CI: 0.3–9.7]), 13.0% studied and worked in their birth country but had had a fellowship or training period abroad (12.7% of women, 13.4% of men, *p* = 0.92, 0.6% [95% CI: 0.0–6.0]), 4.5% both studied and worked abroad (4.6% of women, 4.3% of men, *p* = 1.00, 0.3% [95% CI: 0.0–3.6]), and 2.5% studied abroad but currently worked in their birth country (1.2% of women, 4.7% of men, *p* = 0.012, 3.5% [95% CI: 0.6–6.5]).

### Private life

44.4% of the respondents reported not having children, 55.6% had at least one child. Associations between current professional role and parenthood are in Table S3 in the SM.

The difference in caregiving roles beyond childcare between women and men was minor: 4.1% of women provided full-time care compared to 3.0% of men (*p* = 0.48, 1.1% [95% CI: −2.3; 3.9]). Additionally, 15.4% of women shared caregiving responsibilities, compared to 9.9% of men (*p* = 0.049, 5.5% [95% CI: 0; 10.5]). Further childcare support details are in the SM.

**Fig. 2:**
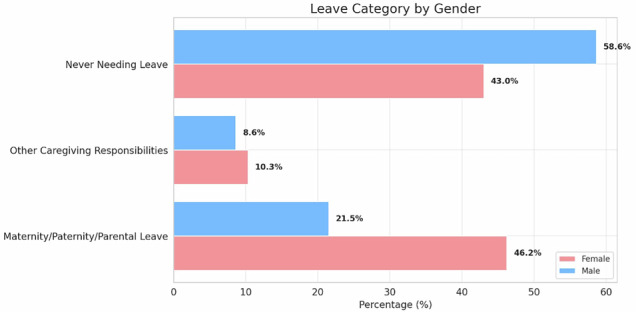
Family-related leaves. The figure shows how many respondents required any kind of leave during their career divided by gender. Parental leave was more common among women (46.2%) than men (21.5%; *p* < 0.001, difference 24.7% [95% CI: 17.3–31.5]). Conversely, more men (58.6%) than women (43.0%) reported never having needed to take leave (*p* < 0.001, difference 15.6% [95% CI: 7.6–23.3])

Family-related leaves were more common and longer for women (46.2% vs 21.5%) (Fig. 2). One third of the respondents reported no difficulties upon returning to work (Fig. S1). However, after a family-related leave, women more frequently reported exclusion from new tasks or projects (13.9% vs 3.9% in men, *p* < 0.001), loss of responsibilities (6.0% vs 0.4%, *p* < 0.001), and difficulty in resuming their previous role (4.6% vs 0.9%, *p* = 0.006), regardless of the duration of absence.

### Timetable and working hours

Full-time work with night shifts was the most reported schedule (43.2%), followed by full-time without night shifts (38.6%). Association with gender and parenthood, and responses from the most represented geographical regions are shown in the SM.

475 out of 648 (73.3%) reported working beyond their official schedule, with similar proportions of those with and without children (55.6% vs 55.5%, *p* = 0.98). Among women, the highest proportion with more than 30% additional working hours was observed in the youngest (≤ 35 years, 41.8%) and oldest (≥ 51 years, 47.4%) age groups. In contrast, men showed the lowest percentage in the youngest group (≤ 35 years, 32.8%), with progressively higher values in older age groups, peaking at 45.9% in those aged ≥ 51 years.

**Fig. 3:**
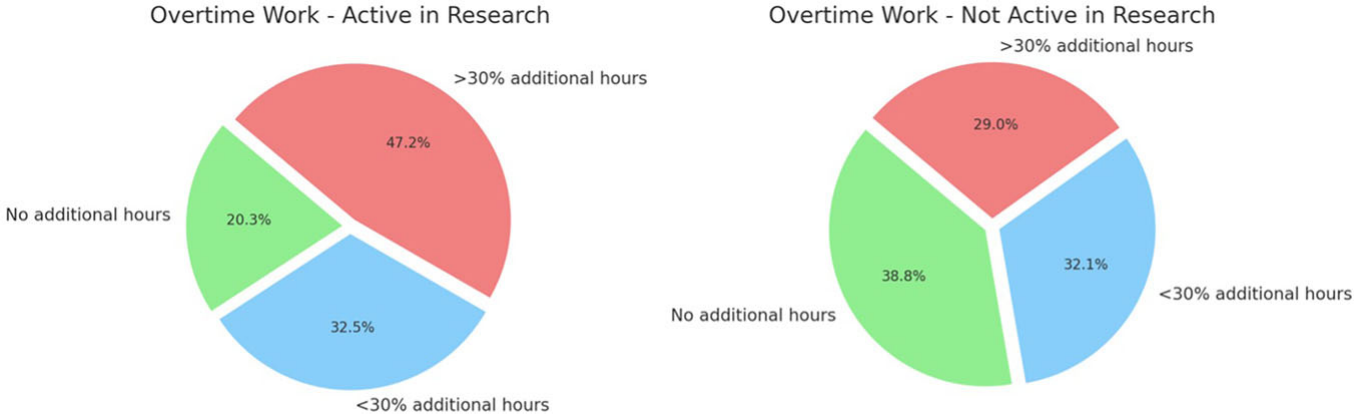
Research activity and overtime work. The pie charts show the frequency of overtime work in respondents participating versus not participating in research activities. Among respondents involved in research, 47.2% reported working more than 30% additional hours beyond their official schedule, compared to 29.0% of those not engaged in research (*p* < 0.001, 18.2% [10.4; 25.5])

Individuals actively engaged in research were more likely to work overtime (Fig. 3). Data on whether the research was conducted or included during working hours are provided in the SM.

### Career

Gender distribution varied across the different specializations, with a higher female representation in diagnostic radiology compared with interventional radiology. Details in SM.

While most respondents (75.5%) stated that gender did not play a role in their choice of subspecialty, 18.3% of women reported that their gender influenced their choice, compared to 1.7% of men (*p* < 0.001, 16.6% [95% CI: 12.3; 20.7]). Most of these women selected breast imaging and pediatric imaging as their subspecialty.

Women were significantly more likely than men to perceive gender as a disadvantage in their careers (44.5% vs 9.5%; *p* < 0.0001 35.0% [95% CI: 28.9 to 41.1]), with variability between regions. Full data are available in the SM.

Reduced possibility to attend on-site conferences (71.8%) was not significantly affected by gender (*p* = 0.24). Limited funding (49.8%) and organizational constraints (48.0%) were the main barriers. No significant geographical region differences were observed (*p* = 1.0). Details are in the SM.

Women published and led grants less than men (data are reported in Tables 2 and 3).

**Table 2:** Publication data

Category	Total *N* (%)	Female *N* (%)	Male *N* (%)
Published at least one article in a peer-reviewed journal	486 (77.8%)	300 (78.1%)	177 (78.3%)
Published in the last 12 months	313 (50.1%)	190 (49.4%)	119 (52.7%)
Published more than 12 months ago	312 (49.9%)	194 (50.6%)	107 (47.3%)
Published as first or last author	459 (73.5%)	275 (71.7%)	173 (76.7%)
Never published as first or last author	165 (26.5%)	109 (28.3%)	52 (23.3%)

The table reports responses related to publication in peer-reviewed journals and authorship, by gender. Men were significantly more likely to have published as first or last authors (*p* = 0.032; difference 8.5% [95% CI: 1.2–15.7]), and this was the only statistically significant difference observed

**Table 3:** Grants acquisition stratified by age groups

Age groups	Have you acquired grants in the last 5 years?	Female *N* (%)	Male *N* (%)
≤ 35	Yes, as PI or co-PI	9 (6.2%)	5 (8.2%)
	Yes, as part of a team	24 (14.4%)	15 (24.6%)
	No	116 (79.5%)	41 (67.2%)
	Total	146 (100.0%)	61 (100.0%)
36–40	Yes, as PI or co-PI	18 (25.0%)	3 (8.8%)
	Yes, as part of a team	12 (16.7%)	6 (17.6%)
	No	42 (58.3%)	25 (73.5%)
	Total	72 (100.0%)	34 (100.0%)
41–50	Yes, as PI or co-PI	20 (19.4%)	16 (30.8%)
	Yes, as part of a team	18 (17.5%)	9 (17.3%)
	No	65 (63.1%)	27 (51.9%)
	Total	103 (100.0%)	52 (100.0%)
≥ 51	Yes, as PI or co-PI	19 (20.0%)	19 (22.4%)
	Yes, as part of a team	16 (16.8%)	13 (15.3%)
	No	60 (63.2%)	53 (62.4%)
	Total	95 (100.0%)	85 (100.0%)
Total	Yes, as PI or co-PI	66 (15.9%)	43 (18.5%)
	Yes, as part of a team	67 (16.1%)	43 (18.5%)
	No	283 (68.0%)	146 (62.9%)
	Total	416 (100.0%)	232 (100.0%)

Grant acquisition increases with age, with younger researchers (≤ 35) having the lowest success rates. Across all age groups, women report a higher percentage of not acquiring grants compared to men (*p* = 0.19, 5.1% [−2.4; 12.8])

### Working environment

Gender distribution of physician staff in the departments is reported in Table 4. Details on the distribution by geographical region are provided in the SM.

**Table 4:** Gender distribution of physician staff and leadership roles within the department

Category	*N* (%)
Department gender composition	
Fairly balanced	259 (39.9%)
Predominantly female (> 60%)	215 (33.2%)
Predominantly male (> 60%)	174 (26.9)
Leadership roles	
Formal position (men)	215 (33.2%)
Formal position (women)	163 (25.2%)
Informal position (men)	115 (17.7%)
Informal position (women)	150 (23.1%)

The majority of departments reported a fairly balanced gender distribution. Formal position refers to a role officially recognized through titles or appointments; informal position is one that is not officially acknowledged by any title or formal nomination. Men were more likely to occupy formal senior positions (33.2%), while women were more likely to occupy informal senior positions (23.1% compared to 17.7% of men, *p* = 0.31, 5.4% [95% CI: −1.0%; 11.8])

A prevalence of male heads of the department (69.9%) was found, while female department heads made up 29.2% (*p* ≤ 0.0001, 40.7% [95% CI: 35.7 to 45.7]). Gender distribution of leadership positions within the department is reported in Table 4. Leadership positions in extracurricular activities (i.e., member of Editorial Board of a peer-reviewed journal, board member of a subspecialty society or task force, etcetera) were predominantly held by men (49.6% vs 40.1%, *p* = 0.025).

**Fig. 4:**
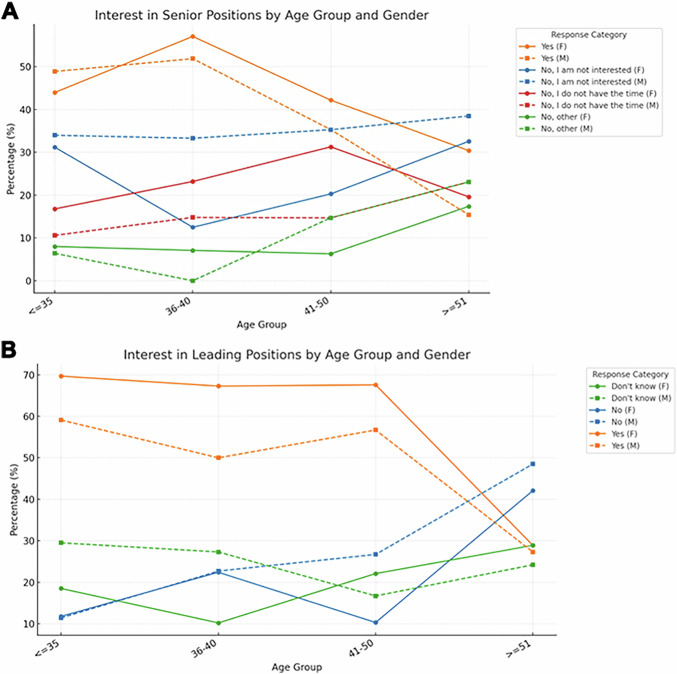
Interest in senior/leading positions. The percentage of people interested in senior positions in the department (**A**) and in leading positions in extracurricular activities (**B**) is presented stratified by age group (*x*-axis) and gender. F, female; M, male

Interest and aspiration in occupying a leading role within the department and/or in extracurricular activities were significantly (*p* < 0.001, 14.4% [95% CI: 7.0 to 21.9]) higher among women and in the youngest age groups, progressively declining with age (Fig. 4).

### Personal experience

**Fig. 5:**
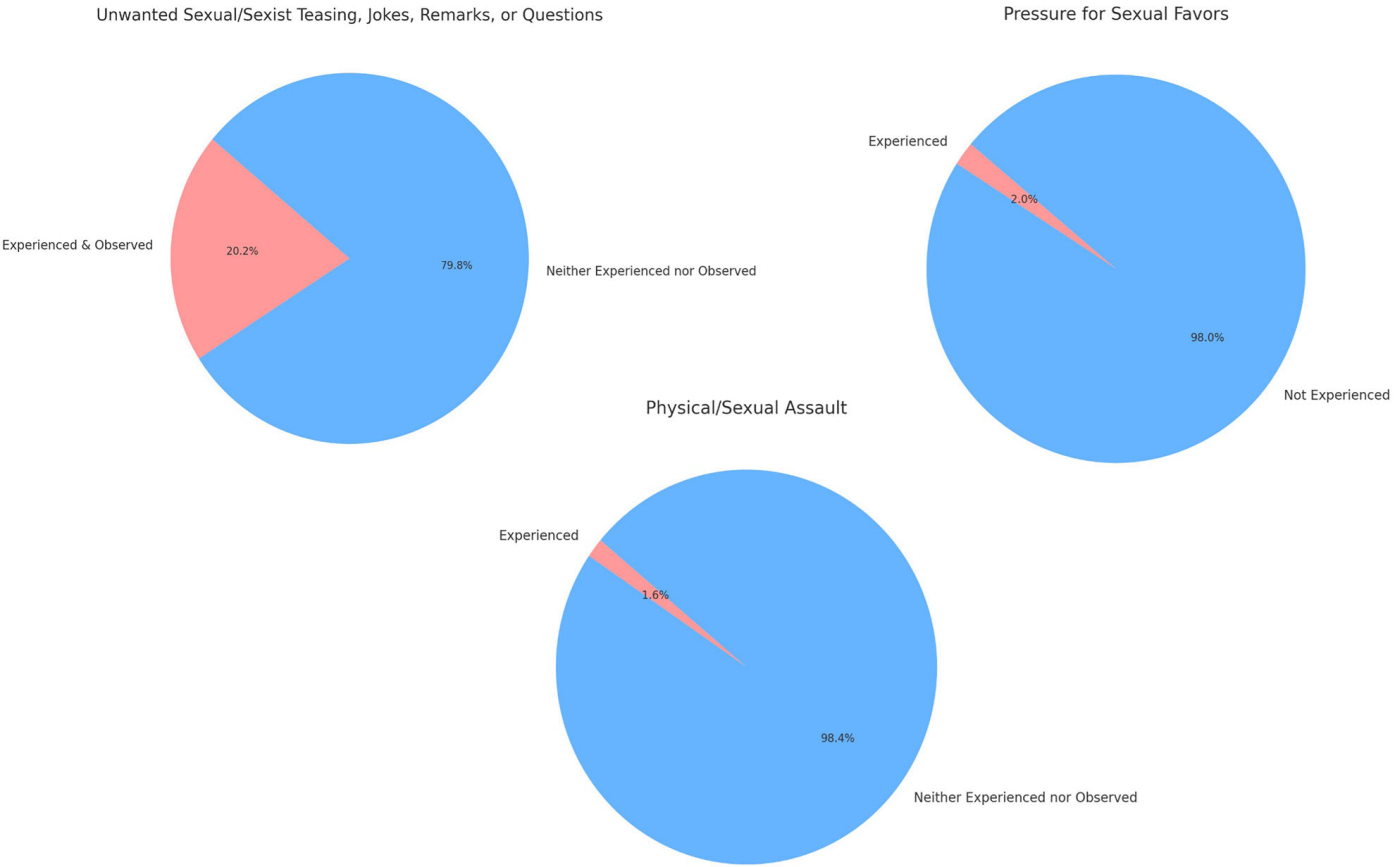
Experience of harassing behavior. The pie charts show how often respondents experienced or observed harassing behavior such as unwanted sexual jokes or remarks, pressure for sexual favors, and sexual assault. Up to 20% of the respondents had “experienced” and/or “observed” forms of harassment or abusive behaviors

A higher percentage of females (57.2%) reported being asked information regarding their private life during job interviews compared to males (31.5%) (*p* < 0.001, 25.7% [95% CI: 17.8; 33.0]). A significantly higher percentage of females (32.2%) reported being addressed in a less respectful manner multiple times compared to males (5.6%) (*p* < 0.001, 26.6% [95% CI: 20.9; 31]), more commonly in Western Europe (*p* = 0.0062). Refer to the SM for the association by geographical regions. In Fig. 5, the data on the experience of harassing behaviors are reported. 1.6% reported experiencing physical/sexual assault.

The perceived career impact of this situation was negative in 19.3% of females compared to 2.4% males (*p* < 0.001, 16.9% [95% CI: 12.4; 21.2]). A positive impact was reported by 0.2% of the total participants, male and female.

**Fig. 6:**
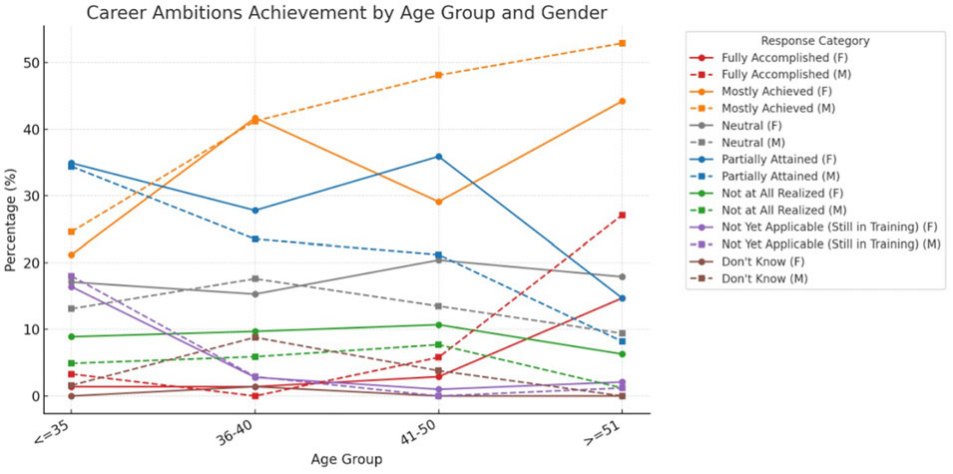
Perceived career achievement by age group and gender. The graphic shows perceived satisfaction with their career achievement of the respondents stratified by age (*x*-axis) and gender

In relation to achievement of personal career ambitions, the responses correlated with age groups and gender (*p* < 0.001) (Fig. 6). Perceived career achievement increased with age in men, while women more often reported only partial fulfillment. Career achievement was more often reported by respondents with children than those without (68.9% vs 45.6%, *p* < 0.0001), without statistically significant gender differences (79.8% vs 46.2%, *p* = 0.0009 for men and 59.4% vs 42.9%, *p* = 0.0029 for women). Family-related leave showed no significant association in career achievement (*p* = 0.20).

## Discussion

The results of this survey underline that, despite progress toward gender equality, issues such as limited organizational support and underrepresentation in leadership continue to impact the professional development of female radiologists.

The gender distribution observed in our respondent population reflects the increasing female representation in the radiological workforce worldwide, in line with the literature [4]. The participants were younger than the general radiological workforce (median age 41 years vs 45% over 51 years) [12]. Most of the respondents came from Europe (68.5%) and Asia (17.1%).

11% of respondents moved abroad after graduating in their birth country, with a slight majority of women, suggesting a greater propensity to seek better career opportunities in more favorable geographical regions. Parenthood rates did not differ significantly by gender, though women showed a tendency to have fewer children. Women showed slightly higher shared caregiving than men. In the context of childcare, women were significantly more likely than men to rely on both informal (partner and family) and formal (paid childcare) support. Parental leave was reported more frequently by women (46.2%) than by men (21.5%), with a higher prevalence among those in leadership positions, clinical radiologists, and academic roles. These findings should be interpreted in light of national policy frameworks. Most countries, except the United States, have implemented paid leave policies that have generally expanded over the past two decades, although substantial cross-national variation remains, influencing economic, social, and health outcomes [13–15]. Recent reforms have extended the duration of paternity leave, which still remains shorter than maternity leave but has shown increased uptake [13].

Only 6.4% of respondents reported shared parenting responsibilities, highlighting the need for cultural and policy changes to promote co-parenting, as women still bear most caregiving duties, despite a gradual move toward more gender-balanced roles [16, 17].

Upon returning to work after a family-related leave, the majority of respondents reported unchanged working conditions; however, significant gender differences emerged in reintegration experiences. Women more frequently reported exclusion from new tasks or projects, loss of responsibilities, and difficulties in resuming their previous role, regardless of the duration of absence. These findings are reflected in the qualitative accounts of female participants, who described being “loaded with work upon return,” having duties “reassigned even before leaving,” and experiencing a “lack of support” at both clinical and personal levels. Similar patterns have been reported in other medical and academic settings [18], highlighting the need for active monitoring and institutional policy action.

Most European participants worked full time with night shifts. Individuals engaged in research were more likely to work extra hours. Moreover, both for male and female research activity was found to be excluded from the job plan in most cases, highlighting the widespread issue of lack of protected time for research activities, in line with both European and non-European literature [19, 20].

Parenthood was not associated with differences in research involvement or in work schedule (full time vs part time). This finding contrasts with the existing literature, where child-rearing is more commonly associated with part-time work [5]. However, among full-time workers, those with children were more likely to perform night shifts. Our study did not explore the reasons behind this pattern, which may vary across settings and relate to night shifts offering greater daytime flexibility, local rota structures, staffing shortages, or financial considerations.

Reported working hours varied widely, reflecting geographical differences and the absence of standardized metrics to define radiology workloads, as previously highlighted [12]. The workforce shortage and organizational constraints were also cited by 48% of our participants as a reason for not attending conferences.

In line with previous reports, our findings confirm persistent gender differences across radiological subspecialties. The reasons behind this underrepresentation appear multifactorial. As shown in prior literature, heavy workloads and challenges in achieving work-life balance are among the main deterrents [4, 21], while the presence of effective mentorship has been recognized as a facilitating factor for women’s engagement in traditionally male-dominated specialties [22, 23]. Radiation exposure concerns, historically cited as a key barrier to entering interventional radiology [4], were rarely mentioned by our respondents, suggesting a possible shift in perceptions due to improved safety standards and greater awareness. Participants stated: “I am planning to have a family and children, so I wanted a good work-life balance,” “I was inspired by my female mentors during residency, as well as by a strong desire to address the unique challenges faced by female patients,” and “I thought being an interventional radiologist would be more difficult as a woman.” It is therefore not surprising that a significant proportion of women, predominantly in Europe, perceive their gender as a disadvantage to their career, in line with similar findings previously reported [4, 24, 25].

Most respondents had published at least one article in a peer-reviewed journal (77.8%), contributing as either first or last author (73.5%); with a higher proportion of men holding first or last authorship roles, with no significant differences between men and women. These findings highlight the increasing involvement and contributions of women in radiology research, particularly over the past two decades [23, 26–29]. Promoting gender parity is crucial, both as a matter of equity and to ensure that research agendas encompass issues pertinent to female populations.

Survey data regarding research funding acquisition align with previous evidence showing increased success with age [30] and no significant gender differences observed.

Our findings on gender distribution among physicians and senior departmental roles align with previous reports [4, 23]. Although many departments report a balanced gender environment, especially in Europe, women remain significantly underrepresented in formal senior roles (25.2% in our findings vs 20% reported in the literature) [23] and in leadership positions within extracurricular activities (40.1%), despite equal expressed interest of both women and men in taking on such roles. For instance, the American College of Radiology (ACR) appointed its first female chair of the Board of Chancellors as late as 2018, and only 13.7% of women are part of editorial boards in radiology journals [26, 27].

Women more frequently received questions about their personal lives during interviews, both in Europe and elsewhere, and report experiencing abuse and disrespectful behavior, in line with previous studies, although earlier research showed higher rates (45–10%) than those observed in our cohort (≤ 20%) [5, 31], highlighting the need for targeted education and awareness to address gender-based disrespect. Taken together, the data ultimately converge on the question of perceived career achievement, suggesting that structural and cultural factors continue to shape women’s experiences and advancement.

Most respondents reported having either mostly (35.8%) or partially (26.1%) achieved their career ambitions, with notable gender differences emerging across age groups. Among younger respondents (≤ 35 years), there were no substantial gender disparities in the proportions reporting ‘mostly achieved’ or ‘partially attained,’ but with increasing age, women reported fewer “mostly achieved” and more “partially attained” outcomes, showing an opposite trend to men, and contrary to literature reports of age-stable patterns [31, 32]. Although women reported greater challenges in balancing work and caregiving responsibilities, having children was not associated with lower career satisfaction, and this was true for both women and men.

This study has several limitations. The predominance of European respondents may limit the generalizability of findings to a global radiology workforce. Voluntary participation may have introduced self-selection bias, with individuals more interested in gender-related issues more likely to respond. Gender differences in family-related leave duration may reflect unmeasured factors (e.g., voluntary vs involuntary absence), potentially biasing return-to-work outcomes. The exclusion of income data may have limited the interpretation of some responses. An additional limitation is the use of an original questionnaire. Nevertheless, this is customary in this type of research, and questions were tested for clarity and understandability before distribution. Lastly, the cross-sectional design offers only a snapshot in time and does not allow for assessment of causal relationships or temporal changes in gender disparities.

In conclusion, gender does influence careers in radiology. Despite progress toward gender equality, limited organizational support and underrepresentation in leadership continue to impact the professional trajectories of female radiologists, contributing to self-imposed career limitations and persistent inequities in career progression and academic recognition. These findings provide a foundation for targeted strategies and policies to promote equity and inclusion.

## Supplementary information

ELECTRONIC SUPPLEMENTARY MATERIAL

## Data Availability

The data are not available for sharing to safeguard individual privacy.

## Abbreviations

CI: Confidence interval
EDI: Equity, diversity and inclusion
ESR: European Society of Radiology
SM: Supplementary Material

## References

[1] RSNA. RSNA’s commitment to diversity, equity and inclusion. Available via https://www.rsna.org/about/rsna-diversity-equity-inclusion. Accessed 20 May 2025

[2] (2023) Commissione Donne Radiologo. Available via https://sirm.org/commissione-dei/. Accessed 20 May 2025

[3] European Cancer Organisation (2024) Inequalities network. Available via https://www.europeancancer.org/topic-networks/inequalities.html. Accessed 20 May 2025

[4] Vernuccio F, Crimì F, Pepe A, Quaia E (2022) Women in radiology: perceived or true barrier? Tomography 8:1881–1884. 10.3390/tomography8040158 35894023 10.3390/tomography8040158 PMC9332462

[5] Fichera G, Busch IM, Rimondini M, Motta R, Giraudo C (2021) Is empowerment of female radiologists still needed? Findings of a systematic review. Int J Environ Res Public Health. 10.3390/ijerph18041542 10.3390/ijerph18041542 PMC7915271 33562881

[6] Gasnier A, Jereczek-Fossa BA, Pepa M et al (2022) Establishing a benchmark of diversity, equity, inclusion and workforce engagement in radiation oncology in Europe—an ESTRO collaborative project. Radiother Oncol. 10.1016/j.radonc.2022.04.011 10.1016/j.radonc.2022.04.011 35461952

[7] European Society of Radiology (ESR). Available via https://www.myesr.org/about/. Accessed 8 Oct 2025

[8] (2018) Erasmus+ FREE Project. Female academic role model empowerment, equality and sustainability at universities in Mediterranean region: towards 2030 Agenda—questionnaire for academics. Erasmus+ Capacity Building in Higher Education; Project No. 598524-EPP-1-2018-1-ES-EPPKA2-CBHE-JP

[9] European Institute for Gender Equality (2022) Gender equality in academia and research GEAR tool step-by-step guide. Available via https://eige.europa.eu/gender-mainstreaming/toolkits/gear. Accessed 25 July 2025

[10] Eslen-Ziya H, Yildirim TM (2022) Perceptions of gendered-challenges in academia: how women academics see gender hierarchies as barriers to achievement. Gender Work Organ 29:301–308. 10.1111/gwao.12744

[11] UNSD. Methodology. Available via https://unstats.un.org/unsd/methodology/m49/. Accessed 20 May 2025

[12] Brady AP, Paulo G, Brkljacic B et al (2025) Current status of radiologist staffing, education and training in the 27 EU member states. Insights Imaging 16:59. 10.1186/s13244-025-01925-7 40088348 10.1186/s13244-025-01925-7 PMC11910488

[13] Nandi A, Jahagirdar D, Dimitris MC et al (2018) The impact of parental and medical leave policies on socioeconomic and health outcomes in OECD countries: a systematic review of the empirical literature. Milbank Q. 10.1111/1468-0009.12340 10.1111/1468-0009.12340 PMC6131347 30277601

[14] Ghazi Sherbaf F, Lin DDM, Yousem DM (2020) Parental leave policy in radiology residency programs: current status. J Am Coll Radiol. 10.1016/j.jacr.2019.12.032 10.1016/j.jacr.2019.12.032 32275902

[15] Spalluto LB, Arleo EK, Lewis MC, Oates ME, Macura KJ (2018) Addressing needs of women radiologists: opportunities for practice leaders to facilitate change. Radiographics. 10.1148/rg.2018180023 10.1148/rg.2018180023 30303802

[16] Brenan M (2020) Women still handle main household tasks in U.S. In: Gallup.com. Available via https://news.gallup.com/poll/283979/women-handle-main-household-tasks.aspx. Accessed 20 May 2025

[17] Miller CC (2020) Young men embrace gender equality, but they still don’t vacuum. The New York Times. https://www.nytimes.com/2020/02/11/upshot/gender-roles-housework.html

[18] Shollen SL (2018) Women’s leader identity development: building a team for the journey. In: Women’s leadership journeys. New York, US: Routledge

[19] Jylhä-Vuorio P, Rinta-Kiikka I, Mäkikangas A, Hirvonen J, Luukkaala T, Arponen O (2025) Factors associated with research activity among radiologists: results of a Nordic survey. Insights Imaging. 10.1186/s13244-025-02108-0 10.1186/s13244-025-02108-0 PMC12554850 41139362

[20] Hames K, Patlas M, Duszak R (2018) Barriers to resident research in radiology: a Canadian perspective. Can Assoc Radiol J. 10.1016/j.carj.2018.03.006 10.1016/j.carj.2018.03.006 30078398

[21] European Society of Radiology (ESR); Cardiovascular and Interventional Radiological Society of Europe (CIRSE) (2019) Interventional radiology in European radiology departments: a joint survey from the European Society of Radiology (ESR) and the Cardiovascular and Interventional Radiological Society of Europe (CIRSE). Insights Imaging. 10.1186/s13244-019-0698-6 10.1186/s13244-019-0698-6 PMC6375097 30758676

[22] Perez YV, Kesselman A, Abbey-Mensah G, Walsh J (2016) A glance at gender-specific preferences influencing interventional radiology selection. J Vasc Interv Radiol. 10.1016/j.jvir.2015.09.009 10.1016/j.jvir.2015.09.009 26723924

[23] Kubik-Huch RA, Vilgrain V, Krestin GP et al (2020) Women in radiology: gender diversity is not a metric—it is a tool for excellence. Eur Radiol. 10.1007/s00330-019-06493-1 10.1007/s00330-019-06493-1 PMC7033068 31802213

[24] The Royal College of Radiologists. Equality and diversity policy. Available via https://www.rcr.ac.uk/about-us/policies/equality-and-diversity-policy/. Accessed 20 May 2025

[25] Bernard C, Pommier R, Vilgrain V, Ronot M (2020) Gender gap in articles published in *European Radiology* and *CardioVascular and Interventional Radiology*: evolution between 2002 and 2016. Eur Radiol 30:1011–1019. 10.1007/s00330-019-06390-7 31506817 10.1007/s00330-019-06390-7

[26] Piper CL, Scheel JR, Lee CI, Forman HP (2016) Gender trends in radiology authorship: a 35-year analysis. AJR Am J Roentgenol 206:3–7. 10.2214/AJR.15.15116 26700331 10.2214/AJR.15.15116

[27] Jalilianhasanpour R, Charkhchi P, Mirbolouk M, Yousem DM (2019) Underrepresentation of women on radiology editorial boards. J Am Coll Radiol 16:115–120. 10.1016/j.jacr.2018.08.017 30340997 10.1016/j.jacr.2018.08.017

[28] Lawler M, Lewison G, Oliver K et al (2023) Gender inequity in cancer research leadership in Europe: time to act. Eur J Cancer. 10.1016/j.ejca.2023.113345 10.1016/j.ejca.2023.113345 37813780

[29] Lawler M, Davies L, Oberst S et al (2023) European groundshot-addressing Europe’s cancer research challenges: a *Lancet Oncology* Commission. Lancet Oncol. 10.1016/S1470-2045(22)00760-4 10.1016/S1470-2045(22)00540-X 36400101

[30] Ross RG, Greco-Sanders L, Laudenslager M, Reite M (2009) An institutional post-doctoral research training program: predictors of publication rate and federal funding success of its graduates. Acad Psychiatry 33:234–240. 10.1176/appi.ap.33.3.234 10.1176/appi.ap.33.3.234 PMC2705878 19574523

[31] Deitch CH, Sunshine JH, Chan WC, Shaffer KA (1998) Women in the radiology profession: data from a 1995 national survey. AJR Am J Roentgenol. 10.2214/ajr.170.2.9456926 10.2214/ajr.170.2.9456926 9456926

[32] Buddeberg-Fischer B, Hoffmann A, Christen S, Weishaupt D, Kubik-Huch RA (2012) Specialising in radiology in Switzerland: still attractive for medical school graduates? Eur J Radiol. 10.1016/j.ejrad.2011.03.011 10.1016/j.ejrad.2011.03.011 21458185

