# Supplementary material for: Gender equity in radiology and radiology research: a survey by the European Society of Radiology
Source: Insights Imaging. 2026 May 6;17:124. doi: 10.1186/s13244-026-02281-w (PMC13149819; doi:10.1186/s13244-026-02281-w)
Supplement: Supplementary file 1 — ELECTRONIC SUPPLEMENTARY MATERIAL [file 13244_2026_2281_MOESM1_ESM.pdf]

Gender Equity in Radiology and Radiology Research: A Survey by the European Society of Radiology

ELECTRONIC SUPPLEMENTARY MATERIAL

General description

Table S1. Complete list of participating countries

Country	N	%
Armenia	4	0.6%
Australia	7	1.1%
Austria	24	3.7%
Belgium	7	1.1%
Brazil	8	1.2%
Canada	4	0.6%
China	4	0.6%
Croatia	7	1.1%
Denmark	13	2.0%
Egypt	9	1.4%
Finland	8	1.2%
France	14	2.1%
Georgia	4	0.6%
Germany	41	6.2%
Greece	28	4.3%
Hungary	5	0.8%
India	29	4.4%
Iran (Islamic Republic of)	4	0.6%
Israel	5	0.8%
Italy	74	11.3%
Latvia	4	0.6%
Mexico	11	1.7%
Netherlands (Kingdom of the)	24	3.7%
Norway	4	0.6%
Pakistan	6	0.9%
Philippines (the)	6	0.9%

Poland	10	1.5%
Portugal	15	2.3%
Republic of Korea (the)	5	0.8%
Romania	14	2.1%
Saudi Arabia	4	0.6%
Serbia	15	2.3%
Slovakia	4	0.6%
South Africa	6	0.9%
Spain	35	5.3%
Sweden	20	3.0%
Switzerland	21	3.2%
Türkiye	18	2.7%
Ukraine	5	0.8%
United Kingdom of Great Britain and Northern Ireland (the)	39	5.9%
United States of America (the)	21	3.2%
Others*	48	

*Others (three respondents or less): Albania, Argentina, Azerbaijan, Belize, Bosnia and Herzegovina, Bulgaria, Canada, Colombia, Costa Rica, Cyprus, Czechia, Ecuador, Ghana, Iraq, Ireland, Jordan, Kazakhstan, Kenya, Kuwait, Libya, Lithuania, Malaysia, Micronesia (Federated States of), Montenegro, Morocco, New Zealand, Nigeria, North Macedonia, Peru, Republic of Moldova, Russian Federation, Saudi Arabia, Singapore, Slovenia, Somalia, Thailand, Tunisia, Uganda, United Arab Emirates, United Republic of Tanzania, Uruguay, Uzbekistan, Vietnam.

Table S2. Participants current role and gender. The table reports the current role and gender distribution of the participants. Where participants indicated more than one role, the position with the highest title was considered.

*Others, including: 4 “Non-binary” (1 Clinical Radiologist, 2 Academic radiologists and 1 Head of the Department/Chair), 3 “Other (please specify)” (2 Clinical Radiologists and 1 Academic Radiologist) and 2 “Prefer not to disclose” (1 Radiologist with a leading role and 1 Head of the Department/Chair)

	Total N (%)	Male N (%)	Female N (%)	Others* N (%)
Radiologist in training/Fellow	116 (17.7%)	31 (13.3%)	85 (20.4%)	0
PhD student	17 (2.6%)	5 (2.2%)	12 (2.9%)	0
Clinical Radiologist	206 (31.3%)	62 (26.7%)	141 (33.8%)	3 (46%)
Academic Radiologist	84 (12.8%)	31 (13.3%)	50 (12%)	3 (46%)
Radiologist with a leading role	175 (26.6%)	71 (30.6%)	103 (24.7%)	1 (15%)
Head of Department/Chair	59 (9%)	32 (13.8%)	25 (6%)	2 (30%)
Total	657 (100%)	232 (100%)	416 (100%)	9 (100%)

Private life

Table S3. Summary of participants with and without children across professional categories, by male and female gender. * Percentages refer to individuals with children over the total in each category (with and without children).

	Total male and female with children N	Total male (with and without children) N	Male with children N (%)*	Total female (with and without children) N	Female with children N (%)*	p value
Radiologist in training/Fellow	19	31	2 (6%)	85	17 (20%)	0.09
PhD student	2	5	0	12	2 (16.6%)	1.00
Clinical Radiologist	114	62	41 (66.1%)	141	73 (51.8%)	0.06
Academic Radiologist	58	31	19 (61.3%)	50	27 (54%)	0.52
Radiologist with a leading role/Head of Department	179	103	83 (80.6%)	128	96 (75%)	0.31

- Across the different categories, no statistically significant gender differences were observed overall.

Private Life: Child-care

Support in child-care was provided (and/or):

- By partner or spouse (70.6%); more frequent among women (74.4%) than men (64.8%; p=0.01, 9.6% [2.3–17.1]).
- By other family members (45.8%); more common among women (51.6%) than men (37.2%; p<0.001, 14.4% [6.4–22.0]).
- Through paid childcare services (35.9%); used significantly more by women (44.6%) than men (22.1%; p<0.001 difference 22.5% [15.1–29.3]).
- Co-parenting: 6.4% (n=23 respondents).

No external support was reported more frequently by men (24.8%) than women (10.7%; p<0.001, 14.1% [8.0–20.6]).)

Figure S1. Difficulties in returning to work after leaves by gender.

35.3% of male and 34.9% of female indicated that their work conditions remained unchanged. A notable gender difference is observed in the feeling of exclusion from new tasks or projects, reported by 13.9% of women compared to 3.9% of men (p<0.001, 10.1 [4.9-15.2]). 6.0% of women stated that responsibilities were taken away from them, whereas 0.4% of men reported the same experience (p <0.001). The inability to return to the previous position was mentioned by 4.6% of women and 0.9% of men (p=0.006). Other types of difficulties were reported by 5.5% of women and 4.7% of men, including being reassigned to less specialized tasks, pressure to return full-time, or inappropriate remarks from supervisors. Other responders mentioned challenges in balancing work and motherhood, or being overloaded with work after returning.

Additionally, a comparison of leave duration revealed that women who reported return-to-work difficulties had, on average, shorter family-related leaves than those who did not (9.9 vs. 11.3 months; p = 0.58), suggesting that these challenges were not associated with the length of absence.

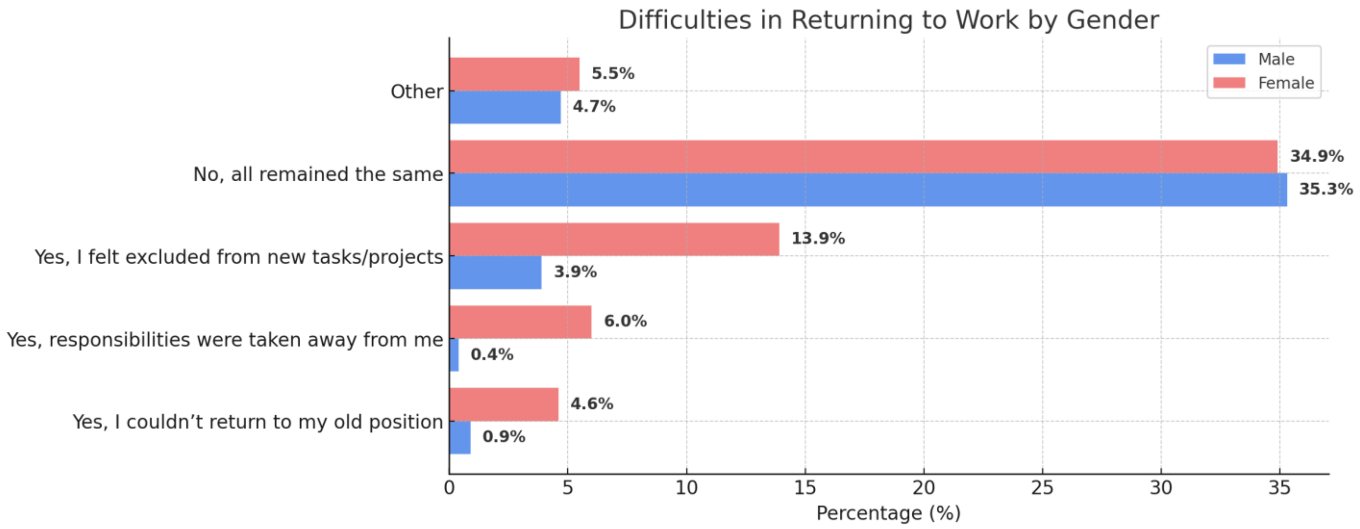

- Regarding family-related leave, most respondents who took such leave also held clinical roles (n = 121), followed by those in leadership positions (n = 105) and academic roles (n = 55). Proportionally, taking family-related leave was more common among academics and leaders. The difference in the distribution of family-related leave across roles was statistically significant ($p < 0.0001$). Among respondents in leadership positions, 70.9% of women reported having taken family-related leave compared to 30.2% of men ($p < 0.0001$). Similar disparities were observed among clinical radiologists (48.5% of women vs. 25.3% of men, $p = 0.0025$). Although women in academic roles also reported higher leave rates than men (48.8% vs. 27.5%), the difference was not statistically significant ($p = 0.071$).

-The association between professional roles and taking family related leave varied significantly across regions ($p<0.0001$). In North and West Europe leave tended to be more concentrated within either clinical or leadership/academic, in South Europe the distribution was more balanced or favored clinical roles, while in East Europe and the Rest of the World family leave was reported almost exclusively among clinical professionals.

Timetable and Working hours

Timetable and Working hours by gender and parenthood:

The majority of respondents work full-time (81.8%).

There was no statistically significant difference between women and men regarding full-time work with or without night shifts (p = 0.40, 3.6% [95% CI: –4.2 to 11.8] and p = 0.17, 6.4% [95% CI: –1.4 to 14.3]), respectively**.** Among full-time workers, 49.6% of those with night shifts had children compared with 38.8% of those without night shifts. This difference was statistically significant (p = 0.009). No significant association was observed between having children and working full-time (55.1%) or part-time (57.6%) (p=0.69).

Timetable and working hours reported by geographical regions:

In Northern and Eastern Europe, the most common arrangement was full-time without night shifts (35.2% and 52.3%), followed by full-time with night shifts (34.1% and 31.8%). Part-time or flexible schedules were less common in these regions, with slightly more women than men choosing part-time work.

In Southern and Western Europe, full-time work with night shifts was the most frequent schedule (51.7% and 52.7%, respectively). In Western Europe, men were more likely than women to work full-time without night shifts (36.2% vs. 19.5%). Comparing European and non-European respondents, European radiologists (68.5%) were more frequently engaged in full-time positions including night shifts (46.4% vs 36.3%, p=0.056), whereas non-European respondents more often worked full-time without night duties (46.6% vs 34.9%, p=0.203).

Research and job plan:

- Among respondents, 54.9% of those active in research and 56.7% of those not active in research had children, with no significant difference between the two groups (p = 0.69).
- 42.7% of respondents performed research outside their job plan, slightly more among men (44.0%) than women (42.1%; p=0.64; difference 1.9% [95% CI: –6.0 to 9.8]).
- 22.4% had research formally included in their job plan, slightly more among men (23.7%) than women (21.6%; p=0.59; difference 2.1% [95% CI: –5.5 to 9.7]).
- 26.5% did not engage in regular research and had no dedicated research time, more commonly reported by women (27.9%) than men (24.1%; p=0.29; difference 3.8% [95% CI: –3.4 to 10.6]).
- 8.3% of respondents were not interested in research, with nearly identical proportions among women (8.4%) and men (8.2%).

Career

Gender distribution and subspecialty:

Breast radiology was more common among female radiologists (22.1%) than their male counterparts (13.4%) (p=0.007, 8.7% [95% CI: 2.5; 14.4]). In contrast, interventional radiology and musculoskeletal radiology were predominantly male-dominated fields, with 17.8% respectively 13.8% of male radiologists specializing in these areas, compared to only 6.5% respectively 5.3% of female radiologists (p<0.001, 11.3% [95% CI: 6.1; 17.1] and p<0.001, 8.5% [95% CI: 3.8; 13.8], respectively). Pediatric radiology was more common among female radiologists (9.6% vs 5.6% males), with no significance difference (p=0.07, 4.0% [95% CI: -0.5; 7.9]).

Participants were asked how their gender influenced their choice of subspecialty. The majority cited a preference for family-friendly specialties (87.5%), while mentorship and role models (0.3%) and perceptions of gender roles (0.2%) were reported much less frequently.

The perceived impact of gender influence on subspecialty choice varied across regions. In Northern and Eastern Europe, 23.1% of women reported gender influence (23.1% and 16.7%, respectively), while men did not. In Southern Europe, 12.7% of women and 1.6% of men reported gender influence, while in Western Europe, the figures were 13.4% for women and 2.1% for men.

When comparing the responses of European and non-European participants, non-European radiologists were more likely to report that gender had influenced their subspecialty choice (15.7% vs 10.8%), whereas European respondents more often indicated that gender had not played a role (78.6% vs 69.6%) (p=0.129).

Perceived influence of gender on career by geographical regions:

- In Southern and Western Europe**,** 51.7% and 52.4% of women considered gender to be “somewhat disadvantageous”.
- In Northern Europe, 41.9% of women perceived gender as “somewhat disadvantageous” to their career, and 6.5% as “highly disadvantageous”. In contrast, only 7.1% of men reported either perception, for both categories (p = 0.039). 73.1% of men considered gender to be neutral compared to only 38.5% of women (p = 0.006).
- In Eastern Europe 50% of women reported a neutral impact, and 6.7% perceived it as “highly disadvantageous”.
- In the Rest of the World, 36.4% of women still perceived gender as somewhat disadvantageous, but 36.4% also described it as neutral.
- Female radiologists in Europe more frequently perceived their gender as disadvantageous to their career (62.7% vs 45.5% outside Europe), whereas non-European women more often described the influence as neutral or advantageous (50.4% vs 34.8%) (p=0.08).

Scientific conference attendance and related barriers:

71.8% of respondents attended fewer than five on-site conferences per year, more commonly among women (73.3%) than men (69.0%) ($p = 0.24$; difference 4.3% [95% CI: –3.0 to 11.7]).

Analyzing the number of on-site conferences attended per year did between European and non-European respondents: overall, 72.1% of European and 71.1% of non-European participants reported attending fewer than five on-site conferences per year ($p=0.785$), while 6.3% and 5.9%, respectively, attended more than ten ($p=1.00$).

Women were slightly more active in presenting research (37.7%) compared to men (32.3%) ($p = 0.17$; 5.4% [95% CI: –2.2 to 13.0]), while rates of invited speakers were similar across genders.

Main barriers to attendance, reported more often by women, included:

- Limited funding (49.8%).
- Organizational constraints (48.0%).
- Family responsibilities (22.7%).

Additional reported obstacles included lack of interest and visa issues (e.g., “Visa is difficult for Iraqi doctor”; “No reimbursement”).

Working environment

Gender distribution of physician staff in the departments, by geographical regions:

In Northern Europe, gender distribution was balanced (51.6%), with similar proportions of female-majority (23.1%) and male-majority (25.3%) units. Eastern Europe had the highest proportion of female-dominated departments (59.1%) while Western Europe showed male-dominated departments (38.8%).

Comparing department gender composition between Europe and non-Europe, European departments were more frequently described as gender-balanced (45.0% vs 29.4%, $p=0.007$), while non-European departments more often had a male predominance (35.8% vs 23.4%, $p=0.002$).

Personal experience

Private life questions asked during job interviews, by geographical regions:

Although some variation exists among European regions, with East Europe showing the highest percentage (57.6%) and South Europe the lowest (44.7%), these differences are not statistically significant ($p = 0.524$).

Questions about private life during job interviews were reported more frequently by women than men, both in Europe (58.7% vs 28.2%, $p<0.001$) and outside Europe (53.1% vs 38.2%, $p=0.044$).

Experiences of harassing behavior, by countries:

Reports of having experienced or observed at least one form of workplace harassment (e.g., sexual remarks, pressure for favors, assault) differ significantly across regions ($p = 0.0062$). The highest rates were observed in Western Europe (47.7%) and Southern Europe (40.8%), while North Europe reported the lowest (15.6%).

QUESTIONNAIRE:

This survey is a key initiative to explore and understand gender dynamics within the field of radiological research. As professionals dedicated to radiology, your insights and experiences are crucial in painting a comprehensive picture of the current state of gender equality in research in our field.

The objectives of this survey are:

1. Understanding the landscape: We aim to gather detailed information on how gender might influence a research career in radiology. This includes aspects such as career progression, workplace interactions, and overall professional experiences.

2. Identifying areas that have room for improvement: Your valuable input will help in pinpointing areas where we, as a community, can strive towards achieving greater gender balance and inclusivity in radiological research.

The survey is aimed at board-certified radiologists, radiologists in training/fellows, and PhD students active in radiological research. Participation in this survey is an opportunity to voice your experiences and to contribute to a significant conversation about fostering an equitable professional research environment. Some questions are personal and sensitive, but they are functional to achieve the objectives of the survey and hopefully will aid in guiding future policies and initiatives within the radiology community.

Your responses will be completely anonymous, and it will not be possible for the authors to identify single participants. The aggregated data will be analysed with the light of the objectives defined above. Care will be taken to not make the data traceable to specific persons or institutions. Relevant results will be submitted for publication in an ESR Journal to inform the community of the current status.

The survey should take approximately 12 minutes to complete. We appreciate your time and honest feedback, which are pivotal in driving positive change in our profession.

Thank you for your valuable contribution.

Data protection, data processing and consent to participate

The ESR Research Committee is responsible for fielding the survey and analysing the survey data. This survey is run and data are collected via the online survey tool SurveyMonkey. All survey data will be reported in aggregate format. Special attention will be paid to ensuring that individuals or specific organisations cannot be traced back in the reported survey results. With the submission of your data/information you agree to these terms. Further information on the protection of your personal data can be found in the ESR privacy notice available at https://www.myesr.org/data-protection-information/

Contact
For questions or remarks on the survey, please contact communications@myesr.org

Demographics

*** 1. Indicate the gender you identify with:**

- ◯ Female
- ◯ Male
- ◯ Non-binary
- ◯ Prefer not to disclose
- ◯ Other (please specify)

*** 2. How old are you?**

*** 3. Where are you based? (*Country*)**

*** 4. Did you study and work in your birth country?**

- ◯ Yes, I studied and work in my birth country
- ◯ I studied in my birth country and then moved abroad
- ◯ Yes, I studied and work in my birth country but did a fellowship (or a period) abroad
- ◯ I studied and I am working abroad
- ◯ I studied abroad but I am currently working in my birth country

*** 5. How would you identify your ethnicity?**

- ◯ Asian or Pacific Islander
- ◯ Black
- ◯ Hispanic or Latino
- ◯ Indigenous (e.g. North American Indian Navajo, South American Indian Quechua, Aboriginal or Torres Strait Islander)
- ◯ Middle Eastern or North African
- ◯ White
- ◯ Prefer not to disclose
- ◯ Other (please specify)

Workplace Environment

* 6. What type of facility do you work in currently? (*Multiple answers possible*)

- ☐ University Hospital / Research Centre
- ☐ Public Hospital
- ☐ Private Hospital
- ☐ Other (please specify)

* 7. What is your timetable?

- ◯ Part time/Flexible with nightshifts
- ◯ Part time/Flexible without nightshifts
- ◯ Full time with nightshifts
- ◯ Full time without nightshifts

* 8. Do you work longer hours than your official schedule?

- ◯ Yes, more than 30% additional hours
- ◯ Yes, less than 30% additional hours
- ◯ Generally, no

* 9. What is your current role? (*Multiple answers possible*)

- ☐ Radiologist in training/Fellow
- ☐ PhD Student
- ☐ Clinical Radiologist
- ☐ Academic Radiologist
- ☐ Radiologist with a leading role (Service chief, head of residency, (Assoc) professor / principal investigator / group leader)
- ☐ Head of Department/Chair

Workplace Environment

* 10. Are you currently active in research?

- ◯ Yes
- ◯ No

* 11. Do you have dedicated time for research in your current job plan?

- ◯ Yes, I do research as a part of my job plan
- ◯ No dedicated time, but I do research outside my job plan
- ◯ No dedicated research time, therefore I do not do research on a regular basis
- ◯ No, I am not interested in doing research

Workplace Environment

* 12. What is your subspeciality? (*Maximum 2 answers*)

- ☐ Breast Radiology
- ☐ Cardiac and Vascular Radiology
- ☐ Chest Radiology/Thoracic Imaging
- ☐ Emergency Radiology
- ☐ Gastrointestinal and Abdominal Radiology
- ☐ Head and Neck Radiology
- ☐ Interventional Radiology
- ☐ Medical Imaging Informatics
- ☐ Musculoskeletal Radiology
- ☐ Neuroradiology
- ☐ Oncologic imaging
- ☐ Paediatric Radiology
- ☐ Urogenital Radiology
- ☐ General Radiology /No specific subspecialty

* 13. Did your gender influence your choice of subspecialty?

- ◯ Yes
- ◯ No
- ◯ Don't know

14. If yes: how did your gender influence your choice of subspecialty?

Workplace Environment

* 15. Which is the gender distribution of physician staff in your *department* ?

- ◯ >60% female
- ◯ >60% male
- ◯ Fairly balanced

* 16. How are the organisational tasks (scheduling, quality assurance/auditing, subgroup leader...) in your department distributed across genders?

- ◯ Fairly balanced
- ◯ Predominantly female
- ◯ Predominantly male
- ◯ Don't know

* 17. What is the gender of your head of department?

- ◯ Female
- ◯ Male
- ◯ Other
- ◯ Prefer to not disclose

Workplace Environment

* 18. Do you have a senior position (responsible for financial, personnel or similar tasks) within your department?

- ◯ Yes, formal senior position
- ◯ Yes, informal senior position (i.e., not recognised in the organisation chart, as a job title, etc.)
- ◯ No

19. If no, would you like to have such a position?

- ◯ Yes
- ◯ No, I am not interested
- ◯ No, I do not have the time
- ◯ No, other

Workplace Environment

* 20. In your research working environment, have you ever been addressed (by patients or colleagues) in a way that seemed less respectful compared to how others in your position are addressed, due specifically to your gender?

- ◯ Yes, multiple times
- ◯ Occasionally
- ◯ Not that I recall

21. In your current research working environment at your place of work have you experienced or observed any of the following behaviours from your colleagues/superiors?

	Experienced	Observed	Experienced and observed	Neither experienced nor observed
Unwanted sexual/sexist teasing, jokes, remarks or questions	◯	◯	◯	◯
Unwanted pressure for dating	◯	◯	◯	◯
Sexual/sexist letters, phone calls, emails	◯	◯	◯	◯
Leaning over, cornering, pinching, touching, non-consensual physical contact	◯	◯	◯	◯
Pressure for sexual favours	◯	◯	◯	◯
Stalking	◯	◯	◯	◯
Physical/sexual assault	◯	◯	◯	◯

22. If you answered any of the questions with experienced and/or observed: do you believe this has impacted your career?

- ◯ No
- ◯ Yes, I was uncomfortable, and the situation had a negative impact on my career
- ◯ Yes, I was uncomfortable, and the situation had a positive impact on my career
- ◯ Yes, I was not uncomfortable, and the situation had a negative impact on my career
- ◯ Yes, I was not uncomfortable, and the situation had a positive impact on my career
- ◯ Not applicable.

Workplace Environment

23. At congresses and conferences , have you experienced or observed any of the following behaviours from your colleagues/superiors?

	Experienced	Observed	Experienced and observed	Neither experienced nor observed
Unwanted sexual/sexist teasing, jokes, remarks or questions	◯	◯	◯	◯
Unwanted pressure for dating	◯	◯	◯	◯
Sexual/sexist letters, phone calls, emails	◯	◯	◯	◯
Leaning over, cornering, pinching, touching, non-consensual physical contact	◯	◯	◯	◯
Pressure for sexual favours	◯	◯	◯	◯
Stalking	◯	◯	◯	◯
Physical/sexual assault	◯	◯	◯	◯

24. If you answered any of the questions with experienced and/or observed: do you believe this has impacted your career?

- ◯ No
- ◯ Yes, I was uncomfortable, and the situation had a negative impact on my career
- ◯ Yes, I was uncomfortable, and the situation had a positive impact on my career
- ◯ Yes, I was not uncomfortable, and the situation had a negative impact on my career
- ◯ Yes, I was not uncomfortable, and the situation had a positive impact on my career
- ◯ Not applicable.

Private Life

* 25. Do you have children?

- ◯ Yes, I have one child
- ◯ Yes, I have more than one child
- ◯ No, I don't have children

26. If you have children, what kind of assistance or support do you receive? (*Multiple answers possible*)

- ☐ Partner/spouse
- ☐ Co-parenting
- ☐ Other family members
- ☐ Childminder/baby-sitter/Nursery (self-paid)
- ☐ Public childcare institutions
- ☐ No support

* 27. Have you ever been asked questions about your private life during a job interview (e.g., Are you in a relationship? Do you have children? Would you like to have children?...)

- ◯ Yes
- ◯ No
- ◯ Don't remember
- ◯ Prefer to not disclose

* 28. Other than children, are you someone's caregiver (e.g., taking care of a person with a disability or an illness)?

- ◯ Yes, I provide the full care
- ◯ Yes, but I share the care tasks
- ◯ No, I am not

Private Life

* 29. Have you taken any family related leaves (e.g., parental leave, maternity/paternity leave, family illness leaves...)? (*Multiple answers possible*)

- [ ] Yes, maternity/paternity/parental leave
- [ ] Yes, leave for other caring responsibilities
- [ ] No, because I did not want to
- [ ] No, because it is not stipulated in my country's legal system
- [ ] No, I never needed to

30. If yes, how many months?

[ ]

31. If yes, did you experience any difficulties in returning to work after this/these leave(s)? (*Multiple answers possible*)

- [ ] Yes, I couldn't return to my old position
- [ ] Yes, responsibilities were taken away from me
- [ ] Yes, I felt excluded from new tasks/projects
- [ ] No, all remained the same
- [ ] Other (please specify)

[ ]

Academic Career

* 32. How many on-site conferences do you attend in a year on average?

- ◯ Fewer than 5
- ◯ 5 to 10
- ◯ More than 10
- ◯ I do not attend on-site conferences

* 33. How many online conferences/courses/webinars do you attend in a year on average?

- ◯ Fewer than 5
- ◯ 5 to 10
- ◯ More than 10
- ◯ I do not attend online conferences/courses/webinars

* 34. If you attend a conference, are you mostly (*Multiple answers possible*):

- ☐ Invited speaker
- ☐ Participant
- ☐ Poster/oral presenter

35. If you are restricted in visiting conferences (you do not attend or attend less than you would like to), what is the reason? (*Multiple answers possible*)

- ☐ Family organisation issues
- ☐ Work organisation issues
- ☐ Not interested
- ☐ Budget/ low workplace reimbursement
- ☐ Other (please specify)

Academic Career

* 36. Have you published at least one article in a peer-reviewed journal?

- ◯ Yes, more than 12 months ago
- ◯ Yes, in the last 12 months
- ◯ No

* 37. Have you ever published articles as first or last author?

- ◯ Yes
- ◯ No

* 38. Do you hold or have you held in the past any leading position in the extracurricular activities within the field (e.g., member of the Editorial Board of a peer-reviewed journal, member of the board of a subspecialty society/taskforce, organisational role of a congress, etc.)?

- ◯ Yes
- ◯ No

39. If not, would you like to?

- ◯ Yes
- ◯ No
- ◯ Don't know

Academic Career

* 40. Have you acquired grants in the last 5 years?

- ◯ Yes, as Principal Investigator (PI) or co-PI
- ◯ Yes, as part of a team
- ◯ No

* 41. Rate the following statements from *strongly disagree* to *strongly agree:*

	Strongly disagree	Disagree	Somewhat disagree	Neutral	Somewhat agree	Agree	Strongly agree
I have sufficient time and opportunities to strengthen my career	◯	◯	◯	◯	◯	◯	◯
I have sufficient time and opportunities to provide excellent care for my patients	◯	◯	◯	◯	◯	◯	◯
I have sufficient time and opportunities to educate students / other radiologists and develop teaching materials	◯	◯	◯	◯	◯	◯	◯
I have sufficient time and opportunities to perform research and drive innovation projects	◯	◯	◯	◯	◯	◯	◯

42. If you are restricted in doing research and acquiring grants what is the main reason (*Multiple answers possible*)?

- ☐ Personal obligations
- ☐ Department organisation / no dedicated time in job plan
- ☐ Clinical workload
- ☐ Not interested
- ☐ Other (please specify)

Academic Career

* 43. If you work in an academic setting, does your university offer programmes (e.g., mentoring, teaching, ...) or career opportunities specifically dedicated to (*Multiple answers possible*):

- [ ] Women
- [ ] Men
- [ ] Minorities
- [ ] All the programs are dedicated to all the personnel
- [ ] I don't know

* 44. How do you perceive the influence of your gender on your career?

- ○ Highly advantageous
- ○ Moderately advantageous
- ○ Neutral
- ○ Somewhat disadvantageous
- ○ Highly disadvantageous
- ○ Don't know

* 45. To what extent do you feel you have achieved your career ambitions?

- ○ Fully accomplished
- ○ Mostly achieved
- ○ Neutral
- ○ Partially attained
- ○ Not at all realised
- ○ Not yet applicable (still in training)
- ○ Don't know
